# The Cilium: Cellular Antenna and Central Processing Unit

**DOI:** 10.1016/j.tcb.2016.08.002

**Published:** 2017-02

**Authors:** Jarema J. Malicki, Colin A. Johnson

**Affiliations:** 1 Bateson Centre and Department of Biomedical Science, University of Sheffield, Western Bank Sheffield, S10 2TN, UK; 2 Wellcome Trust Brenner Building, Leeds Institute of Biomedical and Clinical Sciences, University of Leeds, Beckett Street, Leeds LS9 7TF, UK

**Keywords:** primary cilium, ciliogenesis, cell cycle, actin cytoskeleton, mTOR pathway, autophagy

## Abstract

Cilia mediate an astonishing diversity of processes. Recent advances provide unexpected insights into the regulatory mechanisms of cilium formation, and reveal diverse regulatory inputs that are related to the cell cycle, cytoskeleton, proteostasis, and cilia-mediated signaling itself. Ciliogenesis and cilia maintenance are regulated by reciprocal antagonistic or synergistic influences, often acting in parallel to each other. By receiving parallel inputs, cilia appear to integrate multiple signals into specific outputs and may have functions similar to logic gates of digital systems. Some combinations of input signals appear to impose higher hierarchical control related to the cell cycle. An integrated view of these regulatory inputs will be necessary to understand ciliogenesis and its wider relevance to human biology.

## Trends

Primary cilia integrate complex combinatorial inputs into output functions.

Modulatory inputs arise from the cytoskeleton, proteostasis, and ciliary signaling.

Cell-cycle inputs lead to irreversible disassembly decisions that override other inputs.

The integration of inputs in cilia resembles the behavior of logic gates.

## Ciliogenesis and its Regulation: An Overview

Primary cilia are microtubule-based organelles that are formed from a centriolar anchor, known as the basal body, and extend from the apical surface of most mammalian cells (Box 1). They have evolved to act as a cellular ‘antenna’ that receives diverse signals from the extracellular environment, including light, low molecular weight chemicals, proteins, and mechanical stimuli. Their importance is further highlighted by the studies of a group of diseases, known as ciliopathies, that include cystic kidney disease, neurodevelopmental abnormalities, blindness, obesity, and perhaps even psychiatric disorders 1, 2. Over the past decade the earlier ultrastructural descriptions of cilia (reviewed in [3]) have been consolidated and enriched by new insights into the molecular processes that underlie ciliogenesis or cilia resorption in most, if not all, cells. These include molecular mechanisms that regulate centriole biogenesis and basal body maturation, endomembrane vesicle trafficking, entry into the ciliary compartment through a permeability barrier, and a dedicated protein transport system within the cilium (Box 2). Importantly, cilia include several structural features, in particular those associated with the transition zone, that maintain a fundamental characteristic of the cilium, namely that it functions as a unique subcellular compartment (Box 1).

We attempt here to present an integrative viewpoint of pathways that regulate ciliogenesis and the interrelationships between them. A plethora of recent studies suggest that the regulatory inputs into ciliogenesis can be subdivided into four distinct but interrelated cellular processes, each with an intrinsic logic in their function: the cell cycle, structural influences of the cytoskeleton, cellular proteostasis, and signaling processes.

## Regulatory Inputs from the Cell Cycle

In vertebrates, ciliogenesis and cell division are mutually exclusive because the centrioles must be released from the plasma membrane to function in the mitotic apparatus [4]. Cilia appear to be resorbed throughout G1/S, and evidence has been presented that cilia of a minimal length are found during DNA replication in at least some cell types 5, 6, 7. Cilia are disassembled in two waves: an initial wave occurs before the G1/S transition and a second, major wave occurs before mitotic entry 7, 8. As a consequence, primary cilia are absent throughout mitosis, with only some ciliary membrane attached to the mother centriole [7]. Ciliary resorption then allows centrosomes to contribute to the formation of the main microtubule-organizing centre and mitotic apparatus. Primary cilia reassemble during the G1 phase of the cell cycle and continue to grow as cells exit the cell cycle (G0). Thus, the suppression of ciliogenesis is essential in proliferating cells, and several regulatory pathways coupled to the cell cycle have evolved to prevent inappropriate ciliogenesis.

**Figure 1:**
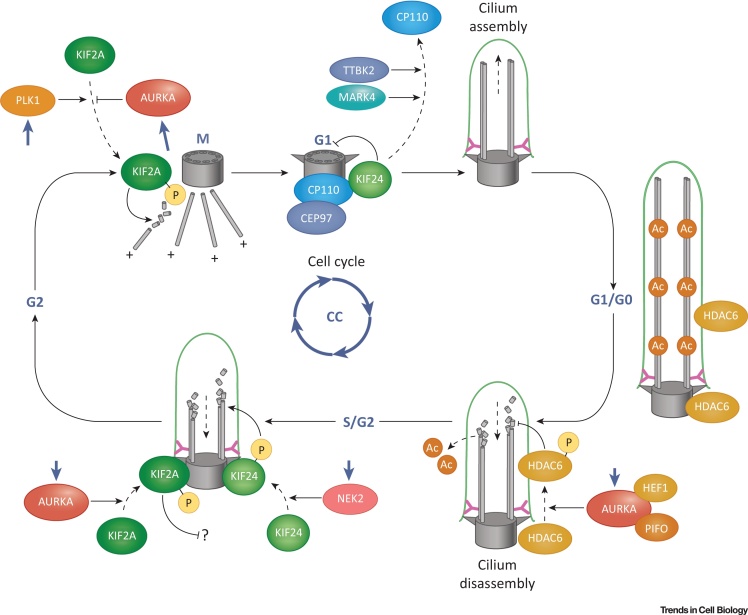
Regulation of Ciliogenesis by Inputs from the Cell Cycle. Cilium length is indicated by axonemal microtubules (grey rods) bound by the ciliary membrane (green line) throughout the cell cycle (CC) in proliferating cells (S, G2, M, and G1 phases), and during exit into and entry from cell quiescence (G0 phase). The mother centriole is indicated by the grey cylinder; only two microtubule doublets are shown for clarity. Stable, acetylated tubulin is indicated at G1/G0 with acetyl groups (Ac) shown as tan circles. The various proteins that are discussed in the main text are highlighted in different colors: the Aurora A (AURKA)–HDAC6 ciliary disassembly pathway is shown in orange and browns, and the kinesin-13 family members (KIF2A and KIF24) that mediate microtubule depolymerization are colored in greens. ‘P’ in a yellow circle indicates protein phosphorylation. Other centrosomal proteins are colored in blues. The role of Kif24 throughout the cell cycle is unclear, and other regulatory proteins are likely to be involved in regulation of the CP110–Cep97 scaffold and phosphorylation by Nek2 during G1/S. AURKA also appears to activate Kif2a during G1/S, but then negatively regulates it by phosphorylation during M. In this and other figures, the shorter arrow-headed lines indicate positive regulatory or activating effects, whereas bar-headed lines indicate negative inhibitory effects. Dashed arrows indicate an inferred physical translocation or post-translational modification of a protein. Purple bold arrows indicate additional regulatory inputs from the cell cycle (‘CC’, circular purple icon).

How are ciliogenesis and cilium disassembly coupled to the cell cycle? Because microtubules are in a state of dynamic equilibrium, one obvious mechanism to regulate ciliogenesis is to alter the balance between the assembly and disassembly of axonemal microtubules. Although the mechanistic details are incompletely understood, several centrosomal and ciliary proteins regulate these processes, and their functions frequently depend upon their phosphorylation by cell-cycle kinases. Examples include Polo-like kinase 4 (PLK4) [9], CP110 and various interacting proteins (CEP97, CEP290, or Talpid3) 10, 11, 12, and the mitochondrial porin and centrosomal protein, VDAC3 [13]. The CP110–Cep97 inhibitory complex [10] is localized at the distal region of the mother centriole (that will subsequently form the basal body; Box 1) and must be inactivated or degraded when cells exit from the cell cycle and form cilia. Protein kinases, such as TTBK2 and MARK4, initiate ciliogenesis by excluding CP110 from the mother centrioles 14, 15 but the exact mechanisms remain unclear. CP110 and Cep97 also play central roles in maintaining a kinesin, Kif24 (a member of the kinesin-13 family), at the distal appendages of the mother centriole to depolymerize centriolar microtubules and therefore suppress ciliogenesis [16]. However, Kif24 also appears to persist at basal bodies after ciliogenesis [16], and its microtubule-depolymerizing activity is induced by interaction with and phosphorylation by Nek2, a NIMA-like S/G2-phase kinase [17]. This process, also now supported by observations in *Chlamydomonas* [18], either blocks the growth of new cilia or suppresses their regrowth after resorption (Figure 1), but the mechanistic details of Kif24 function throughout the cell cycle remain unclear.

Another member of the kinesin-13 family of microtubule depolymerases, Kif2a, that had previously been implicated in the control of mitotic spindle assembly has also been recently shown to function in ciliary disassembly at the mother centriole [19]. Tubulin depolymerization by Kif2a is upregulated by the mitotic Polo-like kinase Plk1, and downregulated by Aurora A [20], a mitotic serine/threonine kinase, which again demonstrates the central role of cell-cycle kinases as ciliogenesis regulators. Plk1 also interacts with and phosphorylates a transition zone ciliopathy protein, nephrocystin-1, thereby integrating a cell-cycle input with the epithelial cell organization and polarity mediated by the nephrocystin protein complex 21, 22. Moreover, the Kif2a–Plk1 pathway appears to be partially redundant to the Kif24–Nek2 pathway and, surprisingly, Kif2a localizes to the subdistal appendages of the mother centrioles which are unlikely to have a direct role in axonemal microtubule depolymerization [19]. One explanation is that Kif2a may instead be depolymerizing centrosomal or cytoplasmic microtubules, thus preventing ciliogenesis outside G0. This could block ciliary assembly by preventing trafficking into the cilium (Figure 1), and may constitute a more general mechanism to integrate regulatory inputs from the cytoskeleton with the cell cycle.

Aurora A is also implicated in a second mechanism of axonemal disassembly in cells emerging from G0. Aurora A associates with HEF1 [8] and Pitchfork (PIFO) [23], and its elevated catalytic activity was reported to induce histone deacetylase 6 (HDAC6) phosphorylation (Figure 1). This stimulates tubulin deacetylation by HDAC6, presumably leading to the destabilization of the ciliary axoneme and cilia resorption [8]. According to several studies, HDAC6 plays a key role in ciliary resorption 8, 24, 25. Nonetheless, the current understanding of this function is clearly incomplete. Notably, *Hdac6* mouse mutants do not have any cilia-related phenotypes [26], suggesting *in vivo* redundancy for HDAC6 function. In addition to acetylation, the α- and β-tubulins of the axoneme are also subject to a diverse range of other post-translational modifications (a ‘tubulin code’ that includes phosphorylation, glycylation, glutamylation, detyrosinylation, and palmitoylation). Although this remains to be investigated, other aspects of the tubulin code are also likely to be cell cycle-regulated. This may be the case for the mitotic spindle-associated protein CEP41, which is required for tubulin glutamylation and the organization of axonemal microtubules [27].

**Figure 2:**
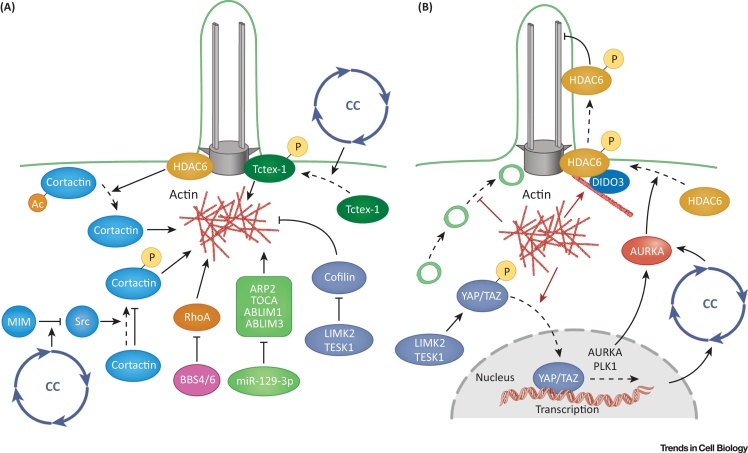
Regulatory Roles of the Cytoskeleton at the Base of the Cilia. (A) Periciliary actin (brown fuzzy lines) is regulated by several pathways, some of which are cell cycle (CC)-dependent. Because BBS proteins have been proposed to form vesicle coats, they may also enhance vesicle access to the cilia base by downregulating actin polymerization. Although this is not indicated in this figure owing to space constraints, many actin polymerization regulators localize to the ciliary base. (B) Actin affects ciliogenesis through at least three distinct mechanisms (brown arrows): (i) regulation of vesicle trafficking to the cilia base; (ii) providing a scaffold to localize ciliogenesis regulators, such as DIDO3/HDAC6; and (iii) through the activation of the YAP/TAZ pathway.

Several regulatory circuits link the cell cycle to both the actin cytoskeleton and ciliogenesis (Figure 2). The activity of cortactin is upregulated through phosphorylation by Src kinase, which appears to be downregulated by MIM (missing in metastasis) during the G1/S phase of the cell cycle [28]. Furthermore, the cytoplasmic dynein light chain protein, Tctex-1, has been reported to facilitate both cell cycle reentry and cilia disassembly [6]. Tctex-1 appears to facilitate actin polymerization, which suggests that actin may mediate its activity both in the cell cycle and in ciliogenesis. Finally, the periciliary actin cytoskeleton as well as two nephrocystins, NPHP4 and NPHP9, appear to regulate the Hippo pathway 29, 30. Because Hippo signaling is an important cell-cycle regulator 31, 32, it provides another link between periciliary actin and cell proliferation.

## Structural Inputs from the Actin Cytoskeleton

Ciliogenesis, and presumably ciliary functions, are modulated by the cytoskeleton. Strong evidence supports the role of actin branching in suppressing primary cilia formation. Actin nucleation-promoting proteins such as cortactin, and components of the ARP2/3 complex, which mediates actin branching, inhibit ciliogenesis 28, 33, 34 (Figure 2). In most studies, actin stress fibers have a similar effect as branched actin networks and inhibit ciliogenesis. Loss-of-function of several ciliary transition zone proteins (including TMEM67, TMEM216, and RPGR) increase stress fiber formation and impair ciliogenesis 35, 36. By contrast, actin-severing factors, such as cofilin and gelsolin-family proteins, have the opposite effect and enhance cilia formation 30, 33. Similarly, a microRNA, miR-129-3p, enhances cilia biogenesis by repressing branched F-actin formation [34].

Actin appears to inhibit ciliogenesis by at least three mechanisms. First, it may have a regulatory role in the transport of cilia-directed vesicles by creating a mechanical barrier to vesicle movement or the membrane remodeling required for ciliogenesis. A role in regulating vesicle transport is supported by observations that actin disassembly results in a transient accumulation of cilia-targeted Smo-positive vesicles at the basal body 30, 33, 34. The dynamics of vesicle accumulation at the cilia base roughly correlates with the transient appearance of Rabin8 and Rab8 in the same area during serum starvation-induced ciliogenesis 30, 37. The actin network could therefore block rapid vesicle transport that is necessary to promote cilium elongation (Figure 2). Second, actin may cause cilia loss by localizing disassembly factors to the cilia base. An example of such a mechanism is DIDO3-mediated HDAC6 targeting to the basal body area [25]. Finally, F-actin polymerization appears to promote cilia disassembly by activating the YAP/TAZ pathway which, in turn, activates Aurora A and, again, HDAC6 [24] (Figure 2). Whereas DIDO3-mediated HDAC6 targeting appears to act locally, the role of YAP/TAZ in ciliogenesis is likely to be mediated at multiple levels through cell-cycle regulation 30, 31. It is also worth noting that HDAC6 activation during cilia disassembly may generate a positive feedback loop by deacetylating cortactin, and further enhancing actin polymerization [38].

Other pathways also mediate F-actin turnover at the cilia base. Bardet–Biedl syndrome (BBS) proteins and the cytoplasmic dynein light chain, Tctex-1, inhibit and enhance actin polymerization, respectively 6, 39 (Figure 2), but because these studies use either inhibitors of RhoA or the actin polymerization inhibitor, cytochalasin D, indirect effects cannot be excluded. Because the centrosome has been recently described to be an actin-organizing center, the actin cytoskeleton could also affect ciliogenesis by influencing basal body docking [40]. In most experimental systems, actin depletion enhances cilia formation, but this is not always the case. In *Chlamydomonas*, inhibitors of actin polymerization shorten flagellae and actin mutants cause a decrease in IFT protein recruitment at the cilia base [41]. Whether similar mechanisms also function in multicellular organisms is unclear.

Cilium interactions with the cytoskeleton are closely related to both planar and apicobasal cell polarity. Cofilin mutants display cilia malpositioned on the apical surface of embryonic node cells, affecting planar cell polarity [42]. In multiciliated cells, both apical actin meshwork and planar subapical microtubules are necessary for translational and rotational polarity of ciliary basal bodies (reviewed in [43]). The cilium localizes to the apical surface in nearly all cells that display apicobasal polarity. This is significant because the apical membrane of the cell is directed towards the lumen of ducts and chambers. As a consequence, it is exposed to mechanical and chemical stimuli different from those at the base of epithelial cells. How basal bodies recognize the apical surface as the docking target remains unclear. Several apicobasal polarity regulators localize to cilia and affect ciliogenesis 44, 45, but evidence that they directly affect basal body docking is lacking. The local remodeling of the actin cytoskeleton that accompanies ciliogenesis will also likely require the regulation of Rho family of small GTPases, as well as adapter proteins, such as Inturned, that couple ciliary proteins to local actin remodeling. However, current evidence for the role of these pathways in ciliogenesis comes from multiciliated cells 46, 47, and it is therefore unclear how they function in primary cilia.

## Cellular Homeostasis and Proteostasis

Viability of the cell depends on the presence of functional proteins in appropriate quantities, maintained through protein synthesis and degradation pathways. There is now compelling evidence that the primary cilium has a role in regulating protein homeostasis by influencing several key protein maintenance mechanisms, including the ubiquitin-proteasome system (UPS), autophagy, and mTOR signaling. The relevance of these three processes to cilia formation and function is discussed below.

**Figure 3:**
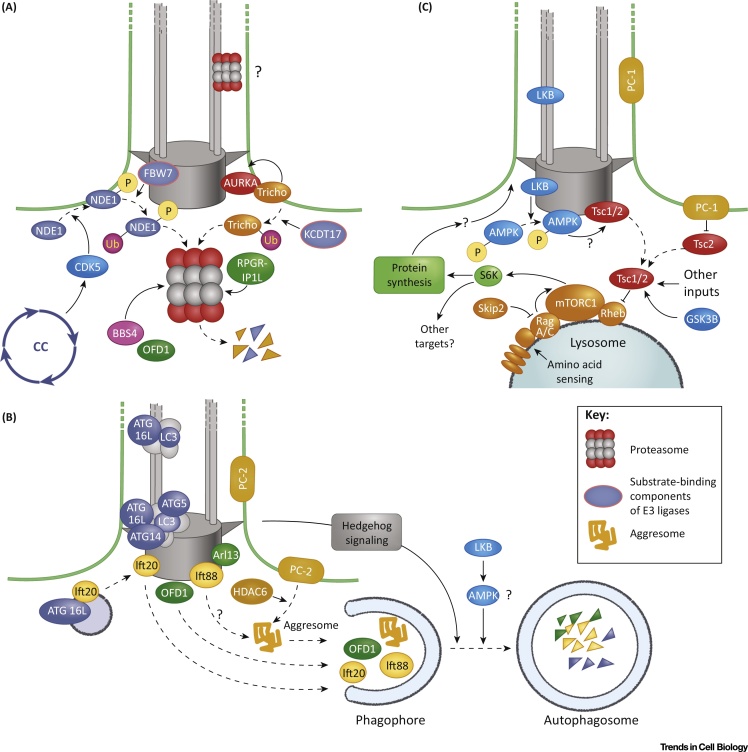
Relationships Between Cellular Proteostasis and Ciliogenesis. (A) Selected UPS-driven regulatory processes in ciliogenesis. At exit from the cell cycle (CC), NDE1 is phosphorylated, which primes it for ubiquitination (‘Ub’ in purple circle). Similarly, trichoplein (Tricho) is ubiquitinated and degraded following cell-cycle exit. RPGRIP1L (also known as MKS5) is a ciliary transition zone protein that directly binds to a regulatory component of the 19S proteasome subunit. (B) Current understanding of functional relationships between the TOR pathway and ciliogenesis. TOR pathway upregulation by the suppression of TSC1/2 or Skip2 function leads to cilia elongation. In a reciprocal mechanism, cilia cause TOR downregulation through LKB and presumably AMPK kinases in response to mechanical stimuli. Although AMPK is known to upregulate TSC2 activity in response to a low-energy state, it is not clear whether it regulates TSC2 in the context of ciliogenesis. TSC1 does localize to the cilia base, and in several experimental contexts its loss-of-function results in cilia elongation. The significance of its ciliary localization is, however, not clear because it functions by activating lysosome membrane-bound Rheb. Loss of cilia as well as polycystin-1 (PC-1) mutations upregulate TOR. Although polycystin-1 upregulates TSC2 by influencing its phosphorylation state, this mechanism may not involve cilia. (C) The current understanding of reciprocal functional relationships between ciliogenesis and autophagy. Following serum withdrawal, autophagy promotes ciliogenesis by eliminating OFD1. By contrast, during sustained serum starvation autophagy appears to downregulate ciliogenesis by eliminating IFT20. Ciliary proteins, such as IFT20 and polycystin-2, enhance autophagy, perhaps by stimulating transport of ATG protein(s) to cilia. Cilia also stimulate autophagy through the Hedgehog pathway. Finally, HDAC6-mediated aggregasome formation may mediate the elimination of damaged ciliary proteins.

The importance of proteasomal degradation in ciliogenesis first came to light based on studies in *Chlamydomonas*. During flagella disassembly, ubiquitination of flagellar proteins increases, and disassembled proteins such as α-tubulin and polycystin-2/PKD-2 are ubiquitin (Ub)-tagged and transported to the cell body by intraflagellar transport [48]. A subsequent study demonstrated that proteosomal degradation affects the stability of proteins (for example, β-catenin, JAG1, GLI2, GLI3, and SUFU) involved in several cilia-related signaling pathways, including Wnt, Notch, and Shh [49]. It also showed that the ciliary/basal body proteins, BBS4 and OFD1, interact with and localize proteasome components to the cilia base (Figure 3A), presumably affecting cilia-mediated signaling. In agreement with this, the inhibition of proteasome function increased cilia length, as demonstrated by using both indirect pharmacological and genetic approaches. Furthermore, several proteasome proteins are found in cilia, and Rpgrip1l, a ciliary transition zone component (Box 1), binds to the regulatory proteasomal subunit Psmd2 [50] (Figure 3A). These and several other studies [51] suggest a close association of the UPS and proteasome function with both cilia formation and cilia-mediated signaling. However, these studies do not distinguish between direct and indirect effector mechanisms, often because of the use of pharmacological agents to infer function, and therefore require some caution in interpretation.

Interestingly, UPS-mediated protein degradation is essential for regulating cilia formation and disassembly during the cell cycle (Figure 3A). Following cell-cycle exit, trichoplein (also known as mitostatin), a negative regulator of ciliogenesis, is degraded by the UPS at the initial step of axoneme extension [52]. Trichoplein prevents ciliogenesis in proliferating cells, after cilia are disassembled at the G0/G1 transition, by binding to and activating Aurora A (see above) at the centrioles [53]. Similarly, at exit from the cell cycle the centrosomal protein NDE1 (another negative regulator of cilia formation) is phosphorylated by a cell cycle-regulated kinase (CDK5), a step that facilitates its recognition by the Ub E3 ligase FBW7 and subsequent degradation during G1/G0 (Figure 3A) [54].

It is important to appreciate that different Ub signals regulate other cellular processes such as endocytosis, for example, that can impinge on ciliogenesis [55]. This is reflected in findings from a recent whole-genome reverse genetics screen, revealing that UPS-dependent ciliogenesis regulators can either enhance (the ANAPC4 subunit of the anaphase-promoting complex) or suppress cilia formation (the E1 Ub-activating enzyme UBE1L/UBA7 and the E2 Ub-conjugating enzyme UBE2I/UBC9) [56]. In addition to ubiquitin, several other Ub-like protein modifiers exist such as SUMO, NEDD8, ISG15, or LC3, but little is known about their potential ciliary functions. In contrast to the Ub system, the small Ub-like modifier (SUMO) system uses the E2 SUMO-conjugating enzyme UBE2I (also known as UBC-9) to recognize and SUMOylate its substrates. Although UBE2I/UBC-9 is a negative regulator of ciliogenesis in mammalian cells [56], in *C. elegans* it is dispensable for cilia formation but ensures the correct ciliary targeting of sensory receptors [57]. This is consistent with the known role of SUMOylation in targeting entire groups of physically interacting proteins for degradation rather than individual polypeptides, particularly in the context of cell-cycle progression [58] and nuclear–cytosolic transport [59]. The latter function may be relevant to similarities between ciliary and nuclear import [60] (Box 1). The role of SUMOylation in cilia will be an interesting future area of research. For example, it will be informative to test if SUMOylation regulates the trafficking of preassembled protein complexes within the ciliary apparatus.

Ciliogenesis is also regulated by the VCP/p97 (hereafter VCP) pathway that separates Ub-labeled proteins from their binding partners and targets them for recycling or degradation by the proteasome. VCP is also involved in autophagosome maturation (reviewed in [61]). In the context of ciliogenesis, UBXN10, a VCP adaptor, binds to the IFT-B complex through CLUAP1/IFT38 (Box 1). UBXN10 localizes to the ciliary axoneme in a VCP-dependant manner and is necessary for cilia formation and maintenance. The function of the VCP/UBXN10 complex in cilia is intriguing: because UBXN10 does not have an obvious Ub-binding activity, it may regulate IFT particle assembly or its interactions with cargo proteins rather than protein turnover [62]. How cargo–IFT particle binding and dissociation are regulated remains largely unknown, and studies on the VCP/UBXN10 complex may offer insights into this process.

Autophagy is another pathway that has recently been shown to function in ciliogenesis. In general, it mediates the non-selective degradation of proteins and organelles such as mitochondria, ribosomes, and peroxisomes to supply amino acids in response to stress conditions such as nutrient starvation 63, 64, 65. Recent evidence demonstrates that autophagy can be selective and resembles the UPS pathway [65]; the lipidated form of the protein LC3 (LC3-II) acts as an Ub-like marker of autophagy, serving as the membrane receptor to select and recruit cargoes for lysosomal degradation [66]. In the context of ciliogenesis, LC3 interacts with a group of centriolar satellite proteins, including PCM1, CEP131, and OFD1 [67]. OFD1 is removed from centriolar satellites upon serum starvation at the initiation of ciliogenesis. In autophagy mutants, such as ATG5, OFD1 degradation is impaired and ciliogenesis inhibited. ATG5 ciliogenesis defects can be rescued by suppressing OFD1 function [67] (Figure 3B). These observations suggest that autophagy of OFD1 is a regulatory step in cilia formation.

The relationship between ciliogenesis and autophagy is, however, more complex because autophagy also appears to limit ciliogenesis by eliminating Ift20, an essential component of ciliary transport [68]. A further level of complexity is added by observations that mutations in Ift20 and Ift88 inhibit serum starvation-induced autophagy, suggesting that at least some IFT proteins are positive regulators of autophagy [68]. To reconcile these observations, Pampliega *et al.* [68] proposed a distinction between basal and induced autophagy, with the latter functioning to enhance ciliogenesis (by removing OFD1, for example) at the onset of serum starvation. By contrast, the basal autophagy limits ciliogenesis by removing Ift20, which provides a potential negative regulatory feedback once cilia are formed (Figure 3B). The signals that target Ift20 and OFD1 to the autophagy pathway appear to have regulatory importance and deserve further investigation. Furthermore, HDAC6 regulates autophagy during autophagosome–lysosome fusion, ensures autophagic processing of ubiquitinated protein aggregates, and therefore also appears to function in autophagy-mediated cilia disassembly through mediating the degradation of ciliary components such as IFT88 [69].

mTOR (mammalian target of rapamycin) complex 1 (TORC1) is a kinase that links amino acid availability to cell growth. It is an important regulator of overall cell metabolism level and a potent inhibitor of autophagy 70, 71. mTORC1 is also a positive regulator of ciliogenesis in mammalian cells [72], a notion supported by findings that mTORC1 signaling positively regulates cilia size in both zebrafish and *Chlamydomonas* through protein synthesis [73] (Figure 3C). Moreover, in at least some experimental conditions, TSC1, a component of the TSC1/2 (hamartin/tuberin) complex that negatively regulates mTOR pathway, has been shown to localize to the basal body, and loss of either TSC1 or TSC2 upregulates ciliogenesis 73, 74, 75. Although this has not been investigated to date, the mTOR pathway could also enhance cilia maintenance by downregulating basal autophagy.

**Figure 4:**
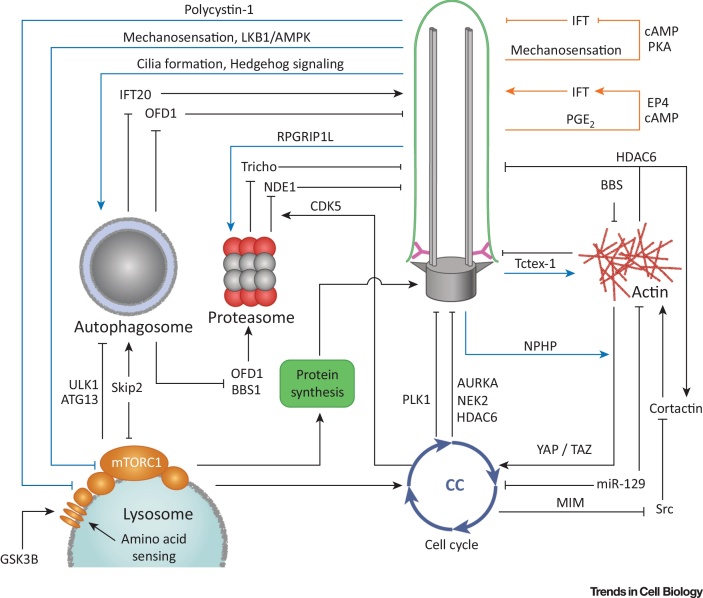
Key Figure: Summary of Ciliogenesis Regulatory Inputs

mTOR signaling may also integrate ciliary mechanosensation with global regulation of cell growth. This is supported by observations that the sensation of flow by renal cilia inhibits both mTORC1 activity and cell size through signaling by a ciliary serine/threonine kinase, LKB1 [76] (Figure 3C). This mechanosensitive pathway also upregulates autophagy, perhaps by inhibiting mTOR (Figure 3C) [77]. Cell size is also regulated by polycystin-1, a ciliary protein that inhibits mTOR through the MEK1/2–ERK1/2 pathway 78, 79. A related protein, polycystin-2, transiently inhibits cilia-dependent autophagy, but not cell size [77]. Because polycystins are also found outside the ciliary compartment, it remains to be determined whether these mechanisms are cilia-related (Figure 3B). Previous studies led to the hypothesis that polycystins act as mechanosensitive calcium channels in the ciliary compartment [80]. Recent data argue, however, against fluctuations in ciliary calcium, implying that the polycystins do not play such a role [81]. The mechanism of ciliary mechanosensation therefore remains a major gap in our knowledge. Finally, in further support of an integrative role for cilia in global cellular proteostasis, interesting but preliminary work using pharmacological approaches has provided indirect evidence that cilia and autophagy regulate reciprocally through the mTOR signaling pathway and the UPS [82]. Thus cilia appear to integrate numerous inputs, ranging from Hedgehog signaling to mechanosensation, that regulate the overall level of cellular metabolism through the mTOR, UPS, and autophagy pathways (Figure 4, Key Figure).

Autophagy is also used for housekeeping purposes such as the degradation of long-lived proteins, elimination of protein aggregates (aggrephagy), and turnover of damaged cellular organelles (mitophagy, ribophagy) 65, 66. mTOR signaling has the opposite effect by limiting autophagy and enhancing protein and lipid synthesis, as well as both glycolysis and oxidative metabolism (reviewed in [83]). The regulatory functions of cilia in mTOR signaling and autophagy thus have wider medical relevance because these pathways are now implicated in many human pathological processes, including neurodegenerative diseases [84] and metabolic disorders [85] such as insulin resistance, obesity, and atherosclerosis. All these processes also affect aging, one of the most pressing problems of affluent societies and a significant challenge to modern biomedical research.

## Modulatory Influences of Cilia-Mediated Signaling on Ciliogenesis

An interesting characteristic of the cilium is its ability to adjust morphology in response to environmental conditions. The enlargement of the cilium to enhance its signal detection capacity or, vice versa, resorption to decrease its responsiveness to signals, makes intuitive sense as mechanisms of sensory signal adaptation. Indeed, cilia appear to act as a self-adjusting antenna, and modify their size and shape depending on the strength of signals that they detect. For example, specialized cilia of *C. elegans* olfactory neurons change morphology from finger-like to fan-shaped in the absence of particular stimuli, and this has been suggested to enhance their olfactory receptivity [86]. A similar mechanism leads to a reduction of cilia length in response to fluid flow, potentially downregulating cilia sensitivity to mechanical stimuli [87]. In these two examples, changes of cilia shape counteract fluctuations in signal intensity, adapting sensory structures to the strength of stimuli. In other contexts, such as leptin or prostaglandin signaling, the presence of the signal enhances ciliogenesis, potentially creating a positive feedback loop 88, 89.

Second messengers and intraflagellar transport play a key role in the adjustment of cilia shape and size in response to extracellular stimuli. Mechanical stimulation of tissue-culture cells decreases cAMP levels and shortens cilia. This is presumably mediated by downregulation of cAMP-dependent kinase signaling and anterograde intraflagellar transport [87]. Similarly, prostaglandin E_2_ (PGE_2_) has been shown to be a positive regulator of ciliogenesis by increasing anterograde intraflagellar transport through its receptor (the GPCR prostaglandin E_2_ receptor 4, EP4) which localizes to the ciliary membrane, and acts by activating the cAMP signaling cascade [88]. Consistent with the importance of cAMP signaling in regulating ciliogenesis, a recent reverse-genetics screen has identified 12 neuroactive GPCRs (including the nociceptin and 5-HT_1B_ serotonin receptors) that localize to the transition zone and mediate ciliogenesis [56]. These receptors may underlie the incompletely understood regulatory mechanisms that modulate cilia formation, and thereby function. Leptin is another extracellular signal that regulates ciliogenesis by modulating intraflagellar transport. It increases cilia length in hypothalamic neurons through the control of the transcription factor RFX1 and upregulation of the expression of IFT-B, but not IFT-A protein genes [89]. This study also implicated F-actin depolymerization (see above) in leptin-mediated cilia length increase, although any effect of altered ciliary signaling on hypothalamic neuronal function and metabolic control was not tested. Finally, phosphatidylinositol (PtdIns) signaling and the cellular ratios of phosphatidylinositol phosphates (PIPs) are implicated in destabilization of the ciliary axoneme, which may have a regulatory role in Hedgehog signaling [90]. The regulation of cilia shape and size by signaling cascades is presumably a modulatory process that occurs in parallel to the regulation of cilia size by cytoskeletal and proteostasis pathways.

## Concluding Remarks and Future Directions

What logic integrates ciliogenesis inputs? The overview of pathways that we have presented above reveals several general themes. First, it appears that interactions of cilia with intracellular processes, such as the cell cycle, autophagy, or cytoskeletal changes involve reciprocal regulatory signaling, presumably providing both positive and negative regulatory feedback loops (Figure 4). For example, downregulation of the mTOR pathway by cilia may then, in turn, limit ciliogenesis, thus maintaining specific cilia length (Figure 4). A positive RPGRIP1L-mediated feedback loop between transition zone formation and both trichoplein and NDE1 degradation may facilitate the fast onset of ciliogenesis by rapidly removing inhibitory factors. Similarly, HDAC6 regulation of cortactin function may provide positive feedback loop that enhances cilia disassembly. Second, as shown with the example of trichoplein and NDE1, multiple parallel mechanisms frequently mediate regulatory interactions between ciliogenesis and an intracellular pathway (Figure 4). Parallel pathways may tune out random fluctuations and make it easier to adjust the function of a particular process over time: autophagy first appears to enhance and then to limit ciliogenesis by eliminating OFD1 and Ift20, respectively. Third, cilia may integrate inputs from multiple intracellular processes to regulate a specific output, thus facilitating coincidence detection. This is similar to the function of logic gates in man-made digital systems. Cell cycle reentry is a good example because it most likely requires signals originating from the cytoskeleton (loss of contact inhibition), metabolism (availability of nutrients), and developmental/tissue repair pathways (Hedgehog, PDGF). Some of these signals (mTOR) enhance ciliogenesis and inhibit cell cycle reentry when not combined with other inputs. As another example, the cilium could moderate the opposing influences of autophagy and mTOR signaling through the Hedgehog pathway 91, 92. We would argue that the logic of this combinatorial signaling represents one of the most important challenges to the ciliogenesis field. Fourth, ciliogenesis appears to be regulated autonomously within the ciliary compartment itself: intraflagellar transport is modulated in response to external stimuli most likely through ciliary cAMP concentration (Figure 4) 87, 88. Finally, some inputs, such as those related to the cell cycle, appear to impose major irreversible transitions such as those that occur during cilium formation and disassembly at entry and exit from cellular quiescence. By contrast, other inputs are modulatory in nature, and seem to ensure the long-term maintenance of both an intact cilium and cellular homeostasis.

Is there a hierarchy to inputs that regulate ciliogenesis? During cellular quiescence, a highly integrated control of multiple inputs may prevent premature and potentially damaging responses to short-term cellular stresses such as random fluctuations in the levels of nutrients, metabolism, cell size, and cytoskeletal deformations. The convergence of multiple inputs in the specific configuration would alter this ‘steady state’ irreversibly, forcing the cell into division. The ensuing disassembly seems to be largely executed by cell-cycle kinases that rapidly phosphorylate centrosomal or ciliary protein substrates, thereby breaking down the very cellular structure that maintains the steady state. Consistent with this logic, the converse also appears to be true: ciliogenesis is induced under conditions of replicative senescence (a stable form of cell-cycle arrest) through the depletion of CP110, the negative regulatory centrosomal protein [93]. This creates a nexus for the integration of modulatory influences that further regulate cellular homeostasis. The cell-cycle inputs therefore appear to be at the top of the hierarchy of regulatory signals for ciliogenesis.

In conclusion, the cellular ‘antenna’, far from being a passive receiver of input signals, is due for an upgrade to the status of a cellular ‘central processing unit’, and perhaps the main one integrating extracellular signaling with the cell cycle and metabolism. We would argue that an integrated view of the many diverse and complex regulatory inputs and output functions is essential for a full understanding of ciliogenesis and its relevance to human health and disease (see Outstanding Questions). Systems-biology approaches, such as affinity proteomics and reverse-genetics screens of cellular phenotypes or expression profiles, will provide powerful new methodologies to explore the logic circuits and regulatory networks for ciliogenesis.

Outstanding Questions What is the structural basis of selective permeability at the ciliary gate?

How do signaling cascades regulate cilia formation and function?

What is the mechanistic basis of ciliary mechanosensation?

What mechanisms regulate the function of ciliary proteins that mediate cilia-specific autophagy?

What underlies the tissue-specific spatial and temporal differences in ciliogenesis and ciliary function?

What other inputs regulate ciliogenesis, and are they organized into a hierarchy?

Is there a logic that underlies the integration of these diverse inputs?

How can integrated pathways of ciliogenesis, cellular homeostasis, and metabolism be utilized for therapeutic purposes?

## Figures and Tables

**Figure I:**
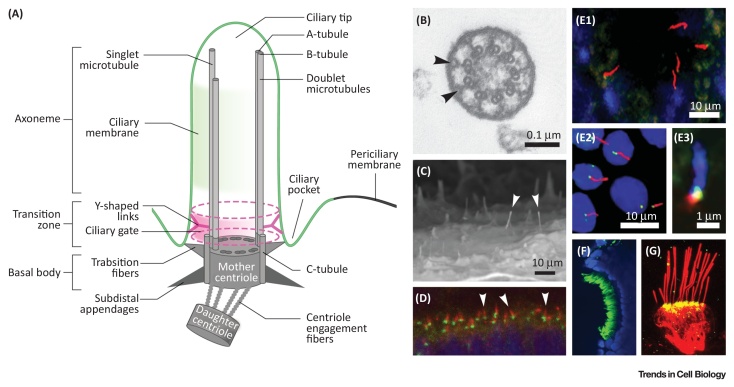
Schematic of Primary Cilia Structure. (A) Simplified schematic of cilium ultrastructure (individual components are not to scale). For the purposes of clarity, the ciliary axoneme is represented by two doublets of microtubules (the A- and B-tubules; grey rods), and the ninefold symmetry is indicated by dark grey ovals in the mother centriole. The axoneme is bound by ciliary membrane (green line and shading). The mother and daughter centrioles are indicated by the grey cylinders, with the third C-tubule extending from the mother centriole towards the ciliary transition zone. The transition zone is also characterized by Y-shaped links (pink) that mediate interactions with the ciliary membrane. Transition fibers (light grey) extend from the distal appendages of the mother centriole. The permeability barrier called the ‘ciliary gate’ is indicated by the dashed pink ovals and pink shading. The ciliary gate is thought to consist of transition fibers, transition zone proteins (reviewed in [95]), and possibly nuclear pore components (such as nucleoporins). (B) Transmission electron micrograph of photoreceptor connecting cilium, showing nine microtubule doublets and Y-shaped links (arrowheads). Reproduced, with permission, from Besharse *et al.* (1985). (C) Scanning electron micrograph of embryonic mouse primary cilia (white arrowheads) on ependymal cells of the lateral ventricle. (D) Immunostaining of photoreceptor cilia and associated basal bodies with anti-IFT88 (red) and anti-γ-tubulin (green) antibodies. (E) Upper panel: human adult kidney collecting duct immunostained for ciliary axoneme (acetylated α-tubulin; red), transition zone (TMEM216; green) and nuclei (DAPI; blue). Left lower panel: ciliated mouse inner medullary collecting duct (mIMCD3) cell line immunostained for acetylated α-tubulin (red), basal body/centrioles (γ-tubulin; green), and nuclei (DAPI; blue). Right lower panel: mIMCD3 cilia stained for acetylated α-tubulin (blue), TMEM216 (green) and γ-tubulin (red). (F,G) Cilia in the nasal placode (F, in green) and ear crista (G, in red) of zebrafish larvae visualized with anti-acetylated α-tubulin antibodies. Specimen in (F) was counterstained with DAPI in blue. Specimen in (G) was double-stained for an apicobasal polarity regulator, Crumbs, in green. Scale bars are as indicated. Images in (D,F,G) are courtesy of N. Pooranachandran and K. Hazime from the laboratory of J.J.M. Images in (C,E) are courtesy of Z.A. Abdelhamed and C.V. Logan from the laboratory of C.A.J.

**Figure II:**
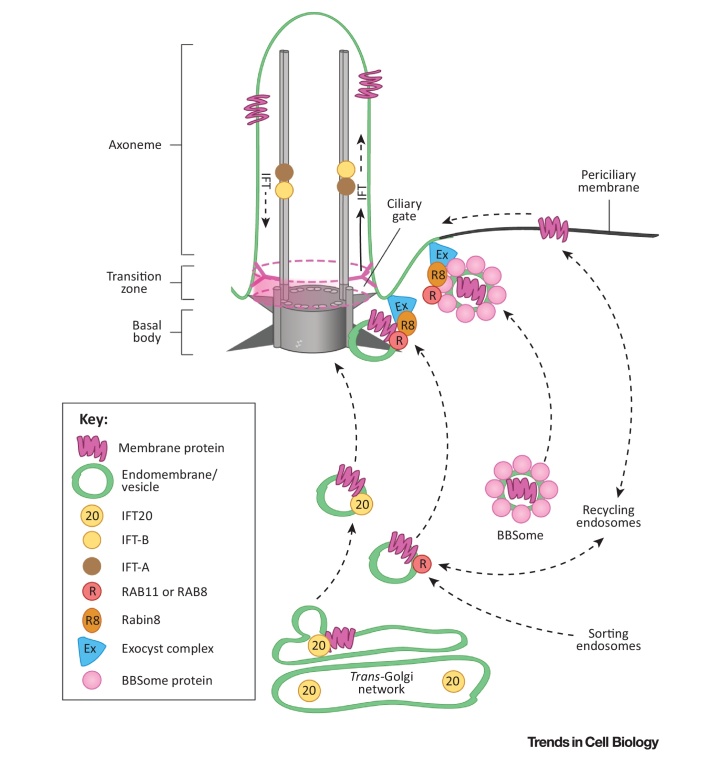
Simplified Schematic of Protein Trafficking Into and Inside the Cilium. Proteins are transported to the base of the cilium by several pathways. This transport step involves BBS proteins, IFT20, and exocyst-related small Rab GTPases. IFT-B (yellow circles) and IFT-A (brown circles) complexes mediate anterograde and retrograde intraflagellar transport (IFT) along the axoneme. Vesicular trafficking and cargo transport are indicated by dashed black arrows. The permeability barrier called the ‘ciliary gate’, that is thought to consist of transition fibers, transition zone proteins (reviewed in [95]), and possibly nuclear pore components (such as nucleoporins), is indicated by the dashed pink ovals and pink shading. Selective trafficking of cargo proteins through the ciliary gate is indicated by the solid black arrow. For details see main text. The key on the left indicates the major protein components and complexes.

## Box 1 Primary Cilia Structure and Function

The main types of cilia have different microtubule structures observed on cross-section (reviewed in [3]). Here we focus on non-motile or ‘primary cilia’ that contain an axoneme, the central cytoskeletal core, consisting of nine microtubule doublets. In quiescent cells the centrosome, consisting of mother and daughter centrioles, migrates to the apical cell surface and matures into the basal body (Figure IA). The centrioles are barrel-shaped, containing nine triplets of microtubules, with each triplet consisting of A-, B-, and C-tubules. The mother centriole then docks to the plasma membrane through fibrous distal and subdistal appendages and becomes the basal body. It acts as a template for microtubule nucleation during ciliogenesis. The axoneme is enclosed by the ciliary membrane that is continuous with the plasma membrane [94]. The distal end of the basal body and a specialized area apical to it function as a permeability barrier between cilium and the cytoplasm, called the ‘ciliary gate’, consisting of two subregions that include transition fibers (TFs) and the transition zone (TZ) [95] (Figure IA). TFs (also known as distal appendages) anchor the mature mother centriole to the plasma membrane through centrosomal proteins such as CEP164 and ODF2. TFs may also form a ‘ciliary pore complex’ that, analogously to the nuclear pore, may confer size-dependent ciliary protein trafficking. The ciliary pocket is a membrane domain at the base of the cilium that is thought to mediate ciliary endocytic activity and vesicular trafficking, and may act as an interface with the actin cytoskeleton. The TZ is distal to the TFs and contains ‘Y-shaped’ linkers 3, 94 (Figure IB). The TZ has a sophisticated modular organization and its components regulate intracellular trafficking to and from the cilium. Many TZ proteins are mutated as causes of ciliopathies [95]. Ciliary cargos pass the ciliary gate and are transported along the axoneme by intraflagellar transport. Intraflagellar transport complexes are thought to assemble at the TFs. Kinesin-2 family motors mediate anterograde transport along the microtubules towards the tip of the cilium by carrying intraflagellar transport subcomplexes A, B, and cargo proteins (see Box 2 figure). Cytoplasmic dynein-2 mediates retrograde movement of intraflagellar transport complexes. Intraflagellar transport mediates both the assembly and resorption of the cilium, and the trafficking of key intermediates of signaling cascades. The ciliary tip limits length and controls structure. It is a regulatory compartment for Gli–Sufu complexes, the effectors of Hedgehog signaling. Examples of cilia from a variety of tissues are shown in Figure IC–G.

## Box 2 Transport to the Cilium

Vesicles containing ciliary proteins bud from the *trans*-Golgi network (TGN) and are directed to a specific docking site at the periciliary base where they tether to the ciliary membrane, facilitated by coordinated interactions between the Rab family – small GTPases related to the exocyst complex (Figure II) [96]. RAB11 and RAB8 mediate vesicular transport to the ciliary base where Rabin8 (the guanine nucleotide exchange factor, RAB8 activator) binds to the exocyst complex protein SEC15. The exocyst then tethers vesicles to the periciliary membrane [97]. CEP290 and CC2D2A are reported to mediate RAB8-dependent membrane-vesicle fusion and trafficking through the ciliary gate [95]. Ciliary protein transport can also be mediated by IFT20 (Figure II). IFT20 may facilitate the packaging of integral membrane proteins such as polycystin-2/PKD2 into vesicles at the TGN and the chaperoning of their transport to the ciliary base. A second pathway also uses the BBSome complex (Figure II). This consists of eight proteins with a coat-like structure and mediates trafficking of integral membrane proteins to the ciliary membrane [98]. The BBSome recognizes putative cilia targeting sequences and is the major effector of Arl6/BBS3 (an Arf-like GTPase). Possible targeting sequences include RVxP (where x is any amino acid) in the N-terminus of polycystin-2 and a C-terminal VxPx sequence in rhodopsin (reviewed in [99]). Rabin8, interacting with the BBSome, regulates RAB8 [98]. The BBSome is not directly required for cilia formation in most tissues, but its failure to deliver ciliary proteins results in defects of cell signaling, embryogenesis, and organogenesis that manifest as a ciliopathy. Following tethering to the ciliary membrane, fusion of the vesicle with the periciliary membrane is presumably facilitated by soluble *N*-ethylmaleimide-sensitive factor receptors (SNAREs) on the vesicle (v-SNARE) and target (t-SNARE) membrane regions [97]. The incorporated membrane proteins then cross the ciliary gate and enter the ciliary membrane, implying active transport for some protein cargos. Some proteins may be then incorporated as intraflagellar transport cargo proteins and transported along the ciliary axoneme. By contrast, passive lateral diffusion from the apical cell membrane into the ciliary membrane has been observed for activated Smoothened. Membrane proteins are prevented from premature exit from cilia by a septin diffusion barrier localized at the base of the primary cilium [100].
